# Exome sequencing revealed *PMM2* gene mutations in a French-Canadian family with congenital atrophy of the cerebellum

**DOI:** 10.1186/2053-8871-1-8

**Published:** 2014-07-04

**Authors:** Anne Noreau, Philippe Beauchemin, Alexandre Dionne-Laporte, FORGE Canada, Patrick A Dion, Guy A Rouleau, Nicolas Dupré

**Affiliations:** Montreal Neurological Institute and Hospital, McGill University, Montreal, Quebec Canada; Department of Pathology and Cellular Biology, Université de Montréal, Montreal, Quebec Canada; Faculty of Medicine of Laval University and the Department of Neurological Sciences of the Centre Hopitalier, Universitaire de Québec, 1401, 18th Street, Quebec, QC G1J 1Z4 Canada; Department of Neurology and Neurosurgery, McGill University, Montreal, Quebec Canada

**Keywords:** Cerebellar, Ataxia, Whole exome sequencing, PMM2, Mutations

## Abstract

Two affected and one unaffected siblings from a French-Canadian family were evaluated in our neurogenetic clinic. The oldest brother had intentional and postural hand tremor while his youngest sister presented mild ataxia, a similar hand tremor and global developmental delay. Brain MRIs of the two affected family members further revealed a significant cerebellar atrophy. For this study we conducted a whole exome sequencing (WES) investigation using genomic DNA prepared from the affected brother and sister, alongside DNA prepared from their unaffected mother, and identified two mutations previously reported to cause a rare disorder known as Congenital Disorder of Glycosylation, type Ia (CDG1A) (OMIM #212065). This study emphasizes how the diagnosis of patients presenting a mild tremor phenotype associated with cerebellar atrophy may benefit from WES in establishing genetic defects associated with their conditions.

## Background

The hereditary cerebellar ataxias are a clinically and genetically heterogeneous group of disorders, where the cerebellum is predominantly affected. The classification of spinocerebellar ataxias has been a subject of debate for several years and in 1988 Harding proposed a first ordering [1]. Since the advent of high-throughput sequencing technologies, prodigious advances have been made in human genetics and the classification and management of hereditary cerebellar ataxias has also been transformed [2]. To date, mutations in numerous genes are known to cause cerebellar ataxias, where S. Jayadev and T.D. Bird recently published a nice overview of hereditary ataxias [3]. Dominant forms can be explained by defects in 35 different genes (SCAs), where many of them are caused by a CAG coding expansion. For the other SCAs, expansions in non-coding regions, point mutations and small insertions/deletions are found. For the recessives forms less genes are known to cause cerebellar ataxia. Indeed, the most common mutated gene is *FRDA*, but mutations in *SACS, POLG, APTX, SYNE1* are also some other gene that can be mutated and cause cerebellar ataxia. Obviously, the presence of specific symptoms can help the clinician to find the proper diagnosis, but it can also become a nightmare. While a whole exome sequencing (WES) approach has been proven to be powerful for the identification of new disease causative genes, the same approach also represents a rapid and efficient tools to screen known genes for the presence of mutations [4]. In Canada, a diagnostic tool using WES was used to diagnose early onset ataxias [5].

## Case presentation

**Figure 1:**
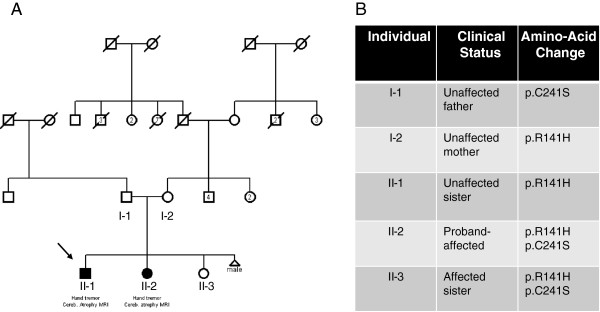
**Pedigree of index Family (A) associated with individual affection status and mutations segregation in the family (B).**

**Figure 2:**
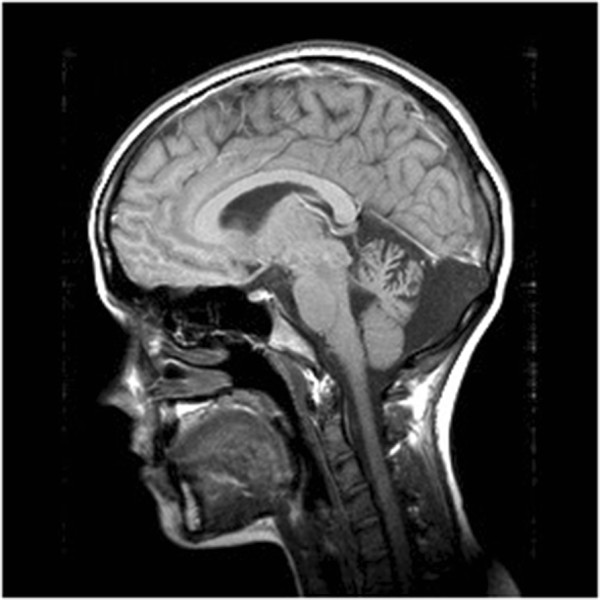
**Sagittal T1 sequence images revealed significant cerebellar atrophy, especially in cerebral vermis in the oldest son.**

Index family: Our family was selected according the FORGE criteria mainly for the presence of a clear recessive mode of inheritance, an early age of onset and at least two affected family members with Canadian origins. This family (Figure 1) was seen at two different time point: for the first time in 2004 and later in 2012, where they underwent clinical evaluation by a neurologist (ND). The proband of the family (arrow, Figure 1-A) was accompanied by his sister and both parents. The father came from the Quebec City and the mother from the Portneuf region, and no consanguinity was known in this family. In 2004, the first case (Figure 1, Individual II-1) was 20 years of age, and was seen for one principal feature: upper limb tremor. This boy was born after a normal pregnancy, normal delivery and with normal APGARS. He walked for the first time at 15 months of age. He was seen in neurology after some language problems that resulted in the failure of his first year at school. At the first neurological examination, the patient was complaining of hand tremor which affected his fine dexterity. He also presented with head tremor. He did not show any dysarthria or ataxia. Magnetic brain imaging (MRI) revealed cerebellar atrophy (Figure 2), localised at the cerebellar hemispheres and the vermis. Her sister (Figure 1, Individual II-2) was 10 years of age at the first neurological examination. Again, the mother reported a normal pregnancy. She was seen early for global developmental delay and hypotonia. She walked for the first time at 23 months of age, with the assistance of orthotics. She had a surgery for alternating strabismus. At a young age, language delay was noted, leading to the necessity to be followed by a speech therapist and by an occupational therapist. At school, she could never attend regular classes. MRI also revealed congenital atrophy of the cerebellum, particularly at vermis. The European Federation of Neurological Societies (EFNS) and European Neurological Society (ENS) recently published guidelines for the diagnosis and management of chronic ataxias [6]. For an autosomal recessive mode of inheritance, found in our family, the recommend a third-step diagnostic approach. The first step is mutation analysis of the *FRDA* gene and biochemical testing, the second step is mutation analysis of *SACS* gene, *POLG*, *APTX* and *SPG7* and the third step is skin or muscle biopsy. In our family, we did mutation analysis for only two most frequent gene known to cause cerebellar ataxia in the French-Canadian population where *SACS* and *FRDA* testing were both negative. Lactic acid and vitamin E levels were also normal. At 18 years old, the most significant clinical features were hand tremor, dysarthria and dysphonia.

Given the growing number of genes involved in cerebellar ataxia, we decided to use a more global approach by using new sequencing technologies. WES was performed on the unaffected mother and two affected children (Figure 1, Individuals I-2, II-2 and II-3). At first a global analysis of sequence coverage was made (data not shown). Hence it was clear that WES produced a good coverage for each of the three individuals. We identified two mutations known to cause a rare disorder called Congenital Disorder of Glycosylation, type Ia (CDG1A) (OMIM #212065). This syndrome is caused by *PMM2* mutations that create a deficiency of the phosphomannomutase (PMM) enzyme which as a subsequent result lead to defective synthesis of N-linked oligosaccharides. In our family, the two affected siblings harbor compound heterozygote mutations: c.422G > A (p.R141H) and c.722G > C (p.C241S) (Figure 1-B). The first mutation (c.422G > A) (p.R141H) was also present in the unaffected sister and this mutation was transmitted by the unaffected mother. On the other hand, the second mutation c.722G > C (p.C241S) was completely absent from the unaffected sister and was clearly transmitted by the father. The two mutations were confirmed by Sanger sequencing, in all individuals available in this family.

## Conclusions

PMM2-CDG is the most common of a group of disorders of abnormal glycosylation of N-linked oligosaccharides. Clinical presentation and course of the disease are highly variable, ranging from infants who die early to mildly involved adults, which is the case for our family [5]. In the infantile multisystem type, infants show axial hypotonia, hyporeflexia, esotropia, developmental delay and language is severely delayed. Hyperglycemia-induced growth hormone release may occur [5], which might explain why the affected daughter is very tall. Some female cases may also lack secondary sexual development [5], which may explain why the affected daughter did not have regular menstrual periods. Since the mutation p.C241A is known to only partially affect the function of the PMM2 enzymes (reduction of activity by only 2 fold), therefore it is in correlation with the mild presentation of the two patients [7–9].

High-throughput sequencing technologies and WES have enabled us to provide a clear genetic diagnosis to explain the condition observed in this family. Given only hand tremor and cerebellar ataxia were observed, it was difficult to establish the exact genetic cause for the disease segregating in this family. With the availability of WES results, the diagnosis became more obvious for the neurologists who saw the patients. Mild ataxic clinical phenotypes have previously been reported in CDG patients, where conventional biochemical analysis results were false negative [10]. For this main reason, we suggest to only perform WES for the diagnosis. Now, it will be possible to better manage the condition of the affected members and also to prevent or follow the onset of other symptoms associated with this syndrome.

## Consent

Written informed consent was obtained from both patients for publication of this Case report. A copy of the written consent is available for review by the Editor-in-Chief of this journal.

## Abbreviations

APTX: Aprataxin gene
DNA: Deoxyribonucleic acid
FRDA: Freidreich ataxia gene (frataxin)
MRI: Magnetic resonance imaging
PMM2: Phosphomannomutase 2 gene
PMM2-CDG: Congenital disorder of glycosylation type 1a
POLG: Polymerase gamma gene
SACS: Spastic ataxia of Charlevoix-Saguenay gene (sacsin)
SCAs: Spinocerebellar ataxias
SYNE1: Spectrin repeat containing, nuclear envelope 1 gene
WES: Whole exome sequencing.
